# Efficacy and safety of dronedarone by atrial fibrillation history duration: Insights from the ATHENA study

**DOI:** 10.1002/clc.23463

**Published:** 2020-10-20

**Authors:** Carina Blomström‐Lundqvist, Nassir Marrouche, Stuart Connolly, Valérie Corp dit Genti, Mattias Wieloch, Andrew Koren, Stefan H. Hohnloser

**Affiliations:** ^1^ Department of Medical Science and Cardiology Uppsala University Uppsala Sweden; ^2^ Section of Cardiology Tulane University Heart and Vascular Institute New Orleans Louisiana USA; ^3^ Population Health Research Institute Hamilton Canada; ^4^ Sanofi‐Aventis Paris France; ^5^ Department of Coagulation Disorders Skåne University Hospital, Lund University Malmö Sweden; ^6^ Sanofi Bridgewater New Jersey, at the time of the study USA; ^7^ Department of Cardiology Division of Clinical Electrophysiology, J. W. Goethe University Frankfurt Germany

**Keywords:** antiarrhythmic therapy, atrial fibrillation, atrial flutter, dronedarone, duration of atrial fibrillation history

## Abstract

**Background:**

Atrial fibrillation/atrial flutter (AF/AFL) burden increases with increasing duration of AF/AFL history.

**Hypothesis:**

Outcomes with dronedarone may also be impacted by duration of AF/AFL history.

**Methods:**

In this post hoc analysis of ATHENA, efficacy and safety of dronedarone vs placebo were assessed in groups categorized by time from first known AF/AFL episode to randomization (ie, duration of AF/AFL history): <3 months (short), 3 to <24 months (intermediate), and ≥ 24 months (long).

**Results:**

Of 2859 patients with data on duration of AF/AFL history, 45.3%, 29.6%, and 25.1% had short, intermediate, and long histories, respectively. Patients in the long history group had the highest prevalence of structural heart disease and were more likely to be in AF/AFL at baseline. Placebo‐treated patients in the long history group also had the highest incidence of AF/AFL recurrence and cardiovascular (CV) hospitalization during the study. The risk of first CV hospitalization/death from any cause was lower with dronedarone vs placebo in patients with short (hazard ratio, 0.79 [95% confidence interval: 0.65‐0.96]) and intermediate (0.72 [0.56‐0.92]) histories; a trend favoring dronedarone was also observed in patients with long history (0.84 [0.66‐1.07]). A similar pattern was observed for first AF/AFL recurrence. No new drug‐related safety issues were identified.

**Conclusions:**

Patients with long AF/AFL history had the highest burden of AF/AFL at baseline and during the study. Dronedarone significantly improved efficacy vs placebo in patients with short and intermediate AF/AFL histories. While exploratory, these results support the potential value in initiating rhythm control treatment early in patients with AF/AFL.

## 1 INTRODUCTION

Atrial fibrillation (AF) is a self‐promoting progressive disease, driven by electrical and structural remodeling processes.^1^, ^2^, ^3^ In comparison to patients with recent‐onset disease, patients who have had AF for an extended period of time will likely have a greater burden of AF. Patients with longer durations of AF history have been shown to have worse outcomes with regard to cardioversion and ablation.^4^, ^5^ The impact of the duration of AF history on the efficacy and safety of commonly used antiarrhythmic drugs (AADs), however, has not been reported.

Dronedarone is an AAD indicated to reduce the risk of hospitalization for AF in patients in sinus rhythm with a history of paroxysmal or persistent AF.^6^ In the ATHENA study (“A Placebo‐Controlled, Double‐Blind, Parallel Arm Trial to Assess the Efficacy of Dronedarone 400 mg BID for the Prevention of Cardiovascular (CV) Hospitalization or Death from any Cause in Patients with AF/AFL (Atrial Flutter)”; NCT00174785), which is one of the largest placebo‐controlled trials of an AAD to date, dronedarone decreased the composite rate of CV hospitalization or death vs placebo in patients with nonpermanent AF or AFL. A total of 4628 patients were randomized in the ATHENA study, with data on duration of AF/AFL history available in over 60% of patients. In this post hoc analysis, we assessed the impact of the duration of AF/AFL history prior to study enrollment on the efficacy and safety of dronedarone.

## 2 METHODS

### 2.1 Overview of the ATHENA study

ATHENA was a double‐blind, placebo‐controlled phase 3 study of 4628 patients with paroxysmal or persistent AF/AFL randomized to dronedarone 400 mg twice daily or placebo in addition to rate‐control therapy; the study design has been described in previous publications.^7^, ^8^ Patients were required to have a 12‐lead electrocardiogram (ECG) within 6 months before randomization indicating AF/AFL and another 12‐lead ECG indicating sinus rhythm during the same period. Initially, patients were included if they were at least 70 years of age or had one of the following CV risk factors: hypertension, diabetes, prior stroke/transient ischemic attack/systemic embolism, left atrial diameter ≥50 mm, or left ventricular ejection fraction <40%. A subsequent amendment to the protocol allowed inclusion of patients ≥75 years old alone or ≥70 years old with at least one of the additional CV risk factors listed above. Patients in AF/AFL at enrollment were expected to undergo cardioversion after appropriate anticoagulation treatment before starting treatment with the study drug. The primary endpoint was the composite of first CV hospitalization or death from any cause. Secondary endpoints included death from any cause, CV death, and first CV hospitalization. Patients were evaluated on days 7 and 14, and months 1, 3, 6, 9, and 12 after randomization, and every 3 months thereafter.

The ATHENA study was conducted in accordance with the Declaration of Helsinki and was approved by independent review boards at participating centers. Patients were enrolled between June 2005 and December 2006 and followed up until December 2007 (minimum follow‐up duration of 12 months).

### 2.2 Post hoc analysis by duration of AF/AFL history

We evaluated patients in the ATHENA study who had investigator‐documented history of first known AF/AFL episode. Duration of AF/AFL history was defined as the time from the first known episode of AF/AFL to randomization, and was subsequently categorized as either short (<3 months), intermediate (3 months to <24 months), or long (≥24 months). Patient demographic and disease characteristics, CV history, and baseline medications were assessed among dronedarone‐ and placebo‐treated patients by duration of AF/AFL history. The impact of the duration of AF/AFL history on clinical outcomes and AF/AFL recurrences was assessed in patients treated with placebo to understand the natural history of the disease. Finally, the efficacy (including first CV hospitalization or death from any cause [composite], first CV hospitalization, death from any cause, first AF/AFL recurrence, and cardioversion rates) and safety (including treatment‐emergent adverse events [TEAEs] defined as events that occurred or worsened during study treatment or within 10 days following the last drug intake, serious TEAEs, and TEAEs leading to permanent discontinuation of study drug) of dronedarone vs placebo were analyzed by duration of AF/AFL history.

First recurrence of AF/AFL was assessed in patients who were in sinus rhythm at baseline.^9^ Assessment of AF/AFL recurrence was based on scheduled 12‐lead ECGs (recorded at 7 and 14 days, 1, 3, and 6 months after randomization, then every 6 months thereafter) and ECGs recorded during unscheduled visits. Each ECG was classified by the investigator as demonstrating AF/AFL or sinus rhythm. Recurrence of AF/AFL was said to occur at the first instance of AF/AFL based on electrocardiography, cardioversion, or hospitalization for AF/AFL.

### 2.3 Statistical analysis

Baseline characteristics were summarized using descriptive statistics. For time‐to‐event outcomes, cumulative incidence functions in each treatment group were calculated using the Kaplan‐Meier method. Hazard ratios (HRs) and 95% confidence intervals (CIs) were estimated using an unstratified Cox regression model with treatment group as the only factor. Inter‐treatment group comparisons were performed using the log‐rank test. For safety analyses, the frequency of patients with TEAEs, serious TEAEs, and TEAEs leading to discontinuation of study drug were summarized using descriptive statistics. Data were analyzed using SAS version 9.4 (Cary, North Carolina).

## 3 RESULTS

Among the 4628 patients randomized in the ATHENA study, 2859 (61.8%) had documentation of first known AF/AFL episode and constituted the study group for the present analysis (1441 of 2301 patients [62.6%] in the dronedarone treatment arm and 1418 of 2327 patients [60.9%] in the placebo arm). Of these patients, 1296 (45.3%), 845 (29.6%), and 718 (25.1%) had short, intermediate, and long AF/AFL histories, respectively. In the short AF history group, 18 patients (0.6%) had an AF/AFL history duration of <2 days and 219 (7.7%) had an AF/AFL history duration of 2 to <7 days. In all, 1942 patients (67.9%) had an AF/AFL history duration of <12 months.

### 3.1 Baseline characteristics

The distribution of patients by age, sex, body mass index, race, and CHA_2_DS_2_‐VASc scores (0‐1, 2‐3, >3) was balanced across AF/AFL history groups and treatment arms (Table 1). Approximately 60% of patients across treatment arms and AF/AFL history groups had CHA_2_DS_2_‐VASc scores of 2 to 3. The presence of CV comorbidities was more frequent at baseline in patients with long vs short AF/AFL histories. While baseline comorbidities were generally balanced between the dronedarone and placebo treatment arms across AF/AFL history groups, there was a numerically higher incidence of patients with left ventricular ejection fraction <35%, chronic heart failure, structural heart disease, and ischemic cardiomyopathy in the placebo arm compared with the dronedarone arm in the short and intermediate AF/AFL history groups.

**TABLE 1:** Patient demographic characteristics, CV history, and medications at baseline

	Short AF/AFL history (<3 mo)		Intermediate AF/AFL history (3 to <24 mo)		Long AF/AFL history (≥24 mo)	
	Dronedarone (n = 670)	Placebo (n = 626)	Dronedarone (n = 416)	Placebo (n = 429)	Dronedarone (n = 355)	Placebo (n = 363)
Age, mean (SD), years	72.5 (9.0)	72.0 (9.6)	70.9 (9.4)	71.8 (9.2)	71.8 (8.8)	71.7 (8.1)
Male, n (%)	320 (47.8)	339 (54.2)	213 (51.2)	237 (55.2)	197 (55.5)	217 (59.8)
BMI, n (%)						
<30 kg/m^2^	453 (67.6)	444 (70.9)	291 (70.0)	282 (65.7)	242 (68.2)	267 (73.6)
≥30 kg/m^2^	217 (32.4)	182 (29.1)	125 (30.0)	147 (34.3)	113 (31.8)	96 (26.4)
Race, n (%)						
White	581 (86.7)	528 (84.3)	350 (84.1)	370 (86.2)	320 (90.1)	319 (87.9)
Asian	45 (6.7)	42 (6.7)	43 (10.3)	38 (8.9)	27 (7.6)	35 (9.6)
CHA_2_DS_2_‐VASc score, n (%)^a^						
0–1	66 (9.8)	85 (13.6)	62 (14.9)	45 (10.5)	45 (12.7)	53 (14.6)
2–3	387 (57.8)	365 (58.3)	251 (60.3)	250 (58.3)	197 (55.5)	215 (59.2)
>3	217 (32.4)	176 (28.1)	103 (24.7)	134 (31.2)	113 (31.8)	95 (26.2)
Baseline AF/AFL,^b^ n (%)^c^	112 (17.6)	90 (15.1)	92 (22.9)	117 (28.6)	97 (29.2)	103 (30.1)
Left atrial diameter, median (range), mm^d^	43.0 (23.0‐68.0)	43.0 (20.0‐75.0)	43.7 (22.3‐72.0)	44.0 (26.8‐72.0)	45.0 (25.4‐68.0)	44.0 (23.0‐67.0)
Left atrial diameter >40 mm, n (%)^d^	429 (64.8)	382 (62.4)	276 (68.0)	304 (72.7)	247 (71.2)	265 (74.2)
LVEF <35%, n (%)^e^	19 (2.9)	29 (4.7)	13 (3.2)	20 (4.8)	20 (5.7)	19 (5.4)
CHF symptoms, n (%)	163 (24.3)	164 (26.2)	101 (24.3)	134 (31.2)	96 (27.0)	94 (25.9)
NYHA class I	58 (8.7)	47 (7.5)	31 (7.5)	35 (8.2)	29 (8.2)	23 (6.3)
NYHA class II	89 (13.3)	99 (15.8)	57 (13.7)	82 (19.1)	55 (15.5)	58 (16.0)
NYHA class III	16 (2.4)	18 (2.9)	13 (3.1)	17 (4.0)	12 (3.4)	13 (3.6)
CV history, n (%)						
Hypertension	589 (87.9)	526 (84.0)	346 (83.2)	378 (88.1)	294 (82.8)	302 (83.2)
Structural heart disease^f^	347 (52.3)	360 (58.3)	222 (54.0)	247 (58.0)	221 (62.8)	228 (63.2)
Tachycardia	221 (33.0)	254 (40.6)	130 (31.3)	139 (32.4)	141 (39.7)	129 (35.5)
Coronary heart disease	175 (26.1)	193 (30.8)	108 (26.0)	117 (27.3)	118 (33.2)	134 (36.9)
Nonrheumatic valvular heart disease	92 (13.7)	88 (14.1)	46 (11.1)	65 (15.2)	62 (17.5)	57 (15.7)
Pacemaker	40 (6.0)	38 (6.1)	32 (7.7)	28 (6.5)	39 (11.0)	55 (15.2)
Ischemic cardiomyopathy	22 (3.3)	32 (5.1)	19 (4.6)	23 (5.4)	17 (4.8)	24 (6.6)
Cardiac valve surgery	13 (1.9)	26 (4.2)	11 (2.6)	13 (3.0)	10 (2.8)	21 (5.8)
Implanted cardioverter defibrillator	8 (1.2)	10 (1.6)	7 (1.7)	4 (0.9)	7 (2.0)	11 (3.0)
Ablation for AF/AFL	6 (0.9)	10 (1.6)	9 (2.2)	12 (2.8)	28 (7.9)	20 (5.5)
Baseline medications, n (%)						
Beta‐blocker (except sotalol)	455 (67.9)	422 (67.4)	296 (71.2)	290 (67.6)	228 (64.2)	260 (71.6)
Oral anticoagulant	374 (55.8)	329 (52.6)	265 (63.7)	293 (68.3)	246 (69.3)	247 (68.0)
Low‐dose aspirin	331 (49.4)	300 (47.9)	170 (40.9)	161 (37.5)	140 (39.4)	141 (38.8)
Calcium antagonist with heart rate‐lowering effect	95 (14.2)	72 (11.5)	72 (17.3)	64 (14.9)	65 (18.3)	53 (14.6)
Digitalis	83 (12.4)	88 (14.1)	60 (14.4)	59 (13.8)	61 (17.2)	60 (16.5)

Abbreviations: AF, atrial fibrillation; AFL, atrial flutter; BMI, body mass index; CHF, chronic heart failure; CV, cardiovascular; LVEF, left ventricular ejection fraction; NYHA, New York Heart Association.

^a^ Derived a posteriori (not included in the primary analysis of the ATHENA study^8^).

^b^ Based on 12‐lead electrocardiogram.

^c^ <3 months: dronedarone n = 637, placebo n = 595; 3 to <24 months: dronedarone n = 402, placebo n = 409; ≥24 months: dronedarone n = 332, placebo n = 342.

^d^ <3 months: dronedarone n = 662, placebo n = 612; 3 to <24 months: dronedarone n = 406, placebo n = 418; ≥24 months: dronedarone n = 347, placebo n = 357.

^e^ <3 months: dronedarone n = 660, placebo n = 611; 3 to <24 months: dronedarone n = 406, placebo n = 420; ≥24 months: dronedarone n = 348, placebo n = 355.

^f^ <3 months: dronedarone n = 664, placebo n = 617; 3 to <24 months: dronedarone n = 411, placebo n = 426; ≥24 months: dronedarone n = 352, placebo n = 361.

Use of medications at baseline was comparable among the groups, irrespective of the duration of AF/AFL history, except for oral anticoagulants and rate‐lowering drugs such as calcium channel blockers and digitalis, which tended to be more common in patients with long vs short AF/AFL histories, and low‐dose aspirin, which tended to be more common in patients with short vs long AF/AFL histories.

### 3.2 Outcomes by duration of AF/AFL history among patients treated with placebo

Among patients treated with placebo, the composite endpoint of CV hospitalization or death from any cause was similar across AF/AFL history groups, occurring in 35.5%, 34.3%, and 38.0% of patients with short, intermediate, and long histories, respectively (Table 2). CV hospitalization, driven by hospitalization for AF/other supraventricular rhythm disorders, was the main contributor to the composite endpoint, occurring in 31.6% of placebo‐treated patients with short AF/AFL history, 32.6% with intermediate history, and 36.1% of patients with long history (Tables 2 and 3); death from any cause occurred in 7.7%, 4.2%, and 5.2% of patients, respectively. Assessment of AF/AFL recurrence was carried out in 1054 placebo‐treated patients who were in sinus rhythm at baseline. The incidence of AF/AFL recurrence was highest in the long AF/AFL history group (62.7%) followed by intermediate (53.8%) and short (41.6%) history groups. The proportion of patients requiring cardioversion during the study period was more than double in the long AF/AFL history group (26.2%) than the short AF/AFL history group (12.3%), with 21.2% in the intermediate AF/AFL history group.

**TABLE 2:** Efficacy of dronedarone vs placebo according to time since first known AF/AFL

Efficacy endpoint, n (%)	Short AF/AFL history (<3 mo)		Intermediate AF/AFL history (3 to <24 mo)		Long AF/AFL history (≥24 mo)	
	Dronedarone (n = 670)	Placebo (n = 626)	Dronedarone (n = 416)	Placebo (n = 429)	Dronedarone (n = 355)	Placebo (n = 363)
CV hospitalization/death from any cause^a^ ^,^ ^b^	196 (29.3)	222 (35.5)	108 (26.0)	147 (34.3)	120 (33.8)	138 (38.0)
HR (95% CI) Log‐rank *P*‐value	0.79 (0.65‐0.96) .02		0.72 (0.56‐0.92).008		0.84 (0.66‐1.07) .15	
CV hospitalization^a^ ^,^ ^b^	172 (25.7)	198 (31.6)	101 (24.3)	140 (32.6)	111 (31.3)	131 (36.1)
HR (95% CI) Log‐rank *P*‐value	0.78 (0.64‐0.96) .02		0.70 (0.55‐0.91) .007		0.82 (0.63‐1.05) .11	
Death from any cause^a^ ^,^ ^b^	42 (6.3)	48 (7.7)	15 (3.6)	18 (4.2)	21 (5.9)	19 (5.2)
HR (95% CI) Log‐rank *P*‐value	0.82 (0.54‐1.24) .34		0.85 (0.43‐1.68) .64		1.13 (0.61‐2.10) .70	
First AF/AFL recurrence^a^ ^,^ ^c^	182 (34.4)	214 (41.6)	129 (41.3)	155 (53.8)	139 (55.6)	158 (62.7)
HR (95% CI) Log‐rank *P*‐value	0.80 (0.65‐0.97) .02		0.67 (0.53‐0.84) .0006		0.81 (0.65‐1.02) .07	
Median time, days	NR	829	747	552	538	373
Cardioversion^b^	66 (9.9)	77 (12.3)	54 (13.0)	91 (21.2)	76 (21.4)	95 (26.2)

Abbreviations: AF, atrial fibrillation; AFL, atrial flutter; CI, confidence interval; CV, cardiovascular; HR, hazard ratio; NR, (median time) not reached.

^a^ Cox regression model.

^b^ All randomized patients.

^c^ Only patients in sinus rhythm at baseline: AF/AFL <3 months (dronedarone, n = 529; placebo, n = 514); 3 to <24 months (dronedarone, n = 312; placebo, n = 288); ≥24 months (dronedarone n = 250; placebo n = 252).

**TABLE 3:** Reasons for first CV hospitalization according to time since first known AF/AFL

Reason for First CV Hospitalization,^a^ n (%)	Short AF/AFL history (<3 mo)		Intermediate AF/AFL history (3 to <24 mo)		Long AF/AFL history (≥24 mo)	
	Dronedarone	Placebo	Dronedarone	Placebo	Dronedarone	Placebo
	(N = 670)	(N = 626)	(N = 416)	(N = 429)	(N = 355)	(N = 363)
Any	172 (100.0)	198 (100.0)	101 (100.0)	140 (100.0)	111 (100.0)	131 (100.0)
AF/other supraventricular rhythm disorders	65 (37.8)	94 (47.5)	41 (40.6)	61 (43.6)	45 (40.5)	68 (51.9)
Worsening CHF, including pulmonary edema or dyspnea of cardiac origin	30 (17.4)	25 (12.6)	13 (12.9)	21 (15.0)	12 (10.8)	15 (11.5)
Stable angina pectoris or atypical chest pain	14 (8.1)	11 (5.6)	8 (7.9)	9 (6.4)	6 (5.4)	7 (5.3)
Syncope	11 (6.4)	6 (3.0)	2 (2.0)	2 (1.4)	3 (2.7)	2 (1.5)
Implantation of a pacemaker, ICD, or other cardiac device	7 (4.1)	8 (4.0)	3 (3.0)	6 (4.3)	8 (7.2)	5 (3.8)
Transcutaneous coronary, cerebrovascular, or peripheral procedure	7 (4.1)	11 (5.6)	8 (7.9)	7 (5.0)	7 (6.3)	4 (3.1)
MI or unstable angina	6 (3.5)	17 (8.6)	7 (6.9)	11 (7.9)	3 (2.7)	9 (6.9)
Major bleeding (requiring ≥2 units of blood or any intracranial hemorrhage)	6 (3.5)	7 (3.5)	5 (5.0)	7 (5.0)	6 (5.4)	5 (3.8)
Blood pressure‐related (hypotension, hypertension; except syncope)	5 (2.9)	6 (3.0)	5 (5.0)	3 (2.1)	3 (2.7)	3 (2.3)
TIA or stroke (except intracranial hemorrhage)	5 (2.9)	6 (3.0)	3 (3.0)	6 (4.3)	12 (10.8)	5 (3.8)
Pulmonary embolism or deep vein thrombosis	5 (2.9)	1 (0.5)	0	1 (0.7)	0	0
CV surgery except cardiac transplantation	3 (1.7)	3 (1.5)	2 (2.0)	4 (2.9)	7 (6.3)	5 (3.8)
Nonfatal cardiac arrest	2 (1.2)	1 (0.5)	0	0	0	1 (0.8)
Atherosclerosis‐related (if not otherwise specified)	2 (1.2)	1 (0.5)	3 (3.0)	1 (0.7)	0	2 (1.5)
CV infection	1 (0.6)	0	2 (2.0)	0	0	0

Abbreviations: AF, atrial fibrillation; AFL, atrial flutter; CHF, chronic heart failure; CV, cardiovascular; ICD, implantable cardioverter defibrillator; MI, myocardial infarction; TIA, transient ischemic attack.

^a^ Data include reasons for first CV hospitalization occurring in more than one patient in any group.

### 3.3 Outcomes associated with dronedarone vs placebo

Across all AF/AFL history groups, dronedarone was generally associated with improved clinical outcomes and efficacy compared with placebo (Table 2). The risk of first CV hospitalization or death from any cause (composite) was lower with dronedarone compared with placebo for patients with short (HR, 0.79 [95% CI: 0.65‐0.96]; *P* = .02) and intermediate (HR, 0.72 [95% CI: 0.56‐0.92]; *P* = .008) AF/AFL histories, with a similar trend in patients with long AF/AFL history (HR, 0.84 [95% CI: 0.66‐1.07]; *P* = .15). This pattern was also observed for rates of CV hospitalization with dronedarone vs placebo in the short (HR, 0.78 [95% CI: 0.64‐0.96]; *P* = .02), intermediate (HR, 0.70 [95% CI: 0.55‐0.91]; *P* = .007), and long (HR, 0.82 [95% CI: 0.63‐1.05]; *P* = .11) AF/AFL history groups. AF/other supraventricular rhythm disorders were the most common reason for first CV hospitalization (43.8% of total cases) in all AF/AFL history groups (Table 3). Death due to any cause was comparable between patients treated with dronedarone or placebo across AF/AFL history groups (Table 2).

The risk of first AF/AFL recurrence was lower with dronedarone compared with placebo for patients with short (HR, 0.80 [95% CI: 0.65‐0.97]; *P* = .02) and intermediate AF/AFL histories (HR, 0.67 [95% CI: 0.53‐0.84]; *P* = .0006), and a similar trend was observed in patients with long history (HR 0.81 [95% CI: 0.65‐1.02]; *P* = .07; Table 2). Median time to first recurrence was prolonged with dronedarone compared with placebo across all AF/AFL groups. The proportion of patients receiving cardioversion during the study period was lower with dronedarone than with placebo across all AF/AFL history groups.

### 3.4 Safety

In both the dronedarone and placebo treatment arms, rates of TEAEs and serious TEAEs were similar across the short, intermediate, and long AF/AFL history groups (Table 4). Treatment‐emergent deaths in the dronedarone and placebo arms were 3.0% vs 2.3% in patients with short AF/AFL history, 0.7% vs 1.6% in patients with intermediate AF/AFL history, and 2.3% vs 1.4% in patients with long AF/AFL history, respectively. Rates of TEAEs leading to permanent discontinuation of study drug were higher with dronedarone vs placebo in all AF/AFL history groups.

**TABLE 4:** Selected TEAEs^a^ and laboratory abnormalities in >2% of patients in any dronedarone‐treated group

Patients, n (%)	Short AF/AFL history (<3 mo)		Intermediate AF/AFL history (3 to <24 mo)		Long AF/AFL history (≥24 mo)	
	Dronedarone (n = 667)	Placebo (n = 619)	Dronedarone (n = 415)	Placebo (n = 428)	Dronedarone (n = 354)	Placebo (n = 363)
Any TEAE	490 (73.5)	430 (69.5)	310 (74.7)	331 (77.3)	277 (78.2)	267 (73.6)
Any serious TEAE^b^	140 (21.0)	138 (22.3)	92 (22.2)	107 (25.0)	72 (20.3)	78 (21.5)
Infections and infestations	42 (6.3)	37 (6.0)	25 (6.0)	21 (4.9)	13 (3.7)	25 (6.9)
Gastrointestinal disorders	26 (3.9)	24 (3.9)	11 (2.7)	9 (2.1)	12 (3.4)	11 (3.0)
Neoplasms	20 (3.0)	21 (3.4)	9 (2.2)	15 (3.5)	10 (2.8)	13 (3.6)
Injury, poisoning, and procedural complications	15 (2.2)	16 (2.6)	14 (3.4)	16 (3.7)	10 (2.8)	6 (1.7)
Respiratory, thoracic, and mediastinal disorders	14 (2.1)	13 (2.1)	4 (1.0)	9 (2.1)	7 (2.0)	10 (2.8)
Renal and urinary disorders	11 (1.6)	13 (2.1)	10 (2.4)	2 (0.5)	4 (1.1)	4 (1.1)
Musculoskeletal and connective tissue disorders	10 (1.5)	15 (2.4)	12 (2.9)	14 (3.3)	5 (1.4)	7 (1.9)
Deaths^c^	20 (3.0)	14 (2.3)	3 (0.7)	7 (1.6)	8 (2.3)	5 (1.4)
TEAEs leading to permanent discontinuation of study drug	85 (12.7)	45 (7.3)	54 (13.0)	35 (8.2)	47 (13.3)	28 (7.7)

Abbreviations: AF, atrial fibrillation; AFL, atrial flutter; TEAE, treatment‐emergent adverse event.

^a^ Events that occurred or worsened during study treatment or within 10 days following the last drug intake.

^b^ Includes serious adverse events leading to death.

^c^ Included deaths that occurred during study treatment or within 10 days following the last drug intake.

The key results of this study are summarized in the Central Illustration.

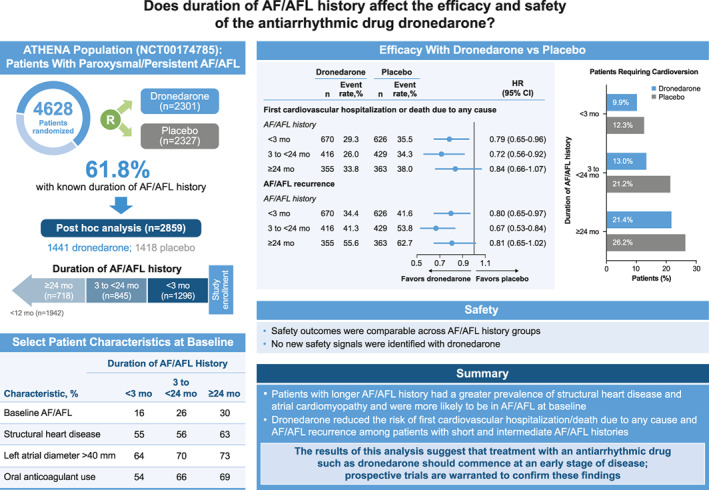

## 4 DISCUSSION

Understanding the importance of the timing of initiating AAD therapy is becoming increasingly relevant, especially with the development of new technologies that have the potential to detect AF at an early stage.^10^, ^11^, ^12^ Early treatment of AF has been shown to benefit patients by reducing the risk of AF progression.^13^, ^14^, ^15^, ^16^, ^17^ Earlier electrical cardioversion, for example, has been shown to reduce time spent in AF, improve symptoms, and delay onset of a subsequent AF episode.^16^ On the other hand, effective treatment may be more difficult in patients with a long AF history, which is associated with a greater disease burden.^4^, ^5^, ^18^ Here, we report for the first time, the efficacy and safety of an AAD in patients with AF/AFL by duration of their disease history. In this analysis, dronedarone significantly reduced the risk of first CV hospitalization or death from any cause (composite) and CV hospitalization, compared with placebo, in patients with short and intermediate AF/AFL histories. As in the primary ATHENA analysis,^8^ there was a trend toward lower relative risk of death due to any cause with dronedarone vs placebo among patients in the short and intermediate AF/AFL history groups, but not in the long AF/AFL history group; however, it should be noted that this post hoc analysis was not adequately powered to assess mortality rates. The rates of first AF/AFL recurrence were also significantly lower with dronedarone vs placebo in the short and intermediate AF/AFL history groups. While not statistically significant, a consistent trend was observed in patients with long AF/AFL history. The incidence of baseline CV comorbidities was slightly higher in the placebo arm vs dronedarone among patients with short and intermediate AF/AFL histories; it is unclear if these differences could have impacted treatment outcomes. Overall, these findings suggest that initiating rhythm control treatment with dronedarone during early stages of AF/AFL may improve clinical outcomes as well as decrease AF/AFL recurrences. However, this hypothesis warrants further investigation.

An association between extended AF/AFL history and AF progression has been reported.^19^ In this analysis, although CHA_2_DS_2_‐VASc scores were similarly distributed across AF/AFL history groups, patients with long AF/AFL history had a greater burden of AF/AFL and other CV comorbidities including left atrial enlargement at baseline, compared with patients with short AF/AFL history. Greater disease burden among placebo‐treated patients in the long AF/AFL history group was also reflected in a higher incidence of AF/AFL recurrence, a shorter time to AF/AFL recurrence, and a higher rate of cardioversions in the long history group compared with the short history group.^9^ Based on the above trends, while the type of AF/AFL (ie, paroxysmal vs persistent) for individual patients at randomization was not collected in the ATHENA trial, we would consider it likely that the short AF/AFL history group was associated with a greater proportion of patients with paroxysmal AF/AFL relative to the intermediate, and especially the long AF/AFL history groups, based on rhythm status at baseline and need for cardioversion during the course of the study. These data suggest that the duration of AF/AFL history can impact patterns of AF/AFL progression, and highlight the importance of taking this aspect into consideration in disease management.

Despite a higher apparent burden of AF/AFL among placebo‐treated patients in the long history group (as reflected in AF/AFL recurrence rates, cardioversion rates, and baseline AF/AFL status), CV hospitalization rate was only modestly greater with increased AF/AFL history duration. These findings may reflect the fact that in comparison to patients with persistent AF, patients with newly diagnosed AF and those with paroxysmal AF typically report more symptoms,^20^ which are associated with higher risk of hospitalization.^21^ Results of CV hospitalization in placebo‐treated patients are also suggestive of the presence of other subacute or unrecognized cardiac conditions, not primarily linked to AF, among patients with recently diagnosed AF/AFL (<3 months).

Safety outcomes, including TEAEs, serious TEAEs, TEAEs leading to permanent discontinuation of study drug, and deaths, were comparable across AF/AFL history groups. The observation that no new safety concerns were identified with dronedarone treatment in any of the AF/AFL history groups is consistent with the overall results of the ATHENA study^8^ and could have important clinical implications from a safety standpoint regarding choice of AADs for treatment.^22^

### 4.1 Limitations

Randomization in the ATHENA study was stratified by baseline AF/AFL status but not by duration of AF/AFL history^8^; therefore, evaluation of clinical outcomes (including CV hospitalization, death, AF/AFL recurrence, and safety) was not powered for such analyses. Owing to this and the post hoc nature of the analysis, these results should be considered exploratory. While patients were categorized into duration of AF/AFL history groups based on time from first known episode of AF/AFL, it is possible that patients could have had prior unidentified episodes. Duration of AF/AFL history was available only in just over 60% of patients in the ATHENA study. Furthermore, information on the overall burden of AF/AFL in patients before randomization was not comprehensive because the length and frequency of episodes as well the characteristics and type of AF/AFL (ie, AF vs AFL, and paroxysmal vs persistent) were not collected. Accurate assessment of AF/AFL recurrence during the study was limited by recording of ECG only during visits to the study center, which was standard during the time of the ATHENA trial, vs continuous monitoring.

## 5 CONCLUSIONS

Duration of AF/AFL history is an important consideration in the evaluation and management of AF/AFL with regard to disease burden as well as response to therapy. The observations of this post hoc analysis of the ATHENA trial suggest that while dronedarone demonstrated efficacy vs placebo regardless of the duration of AF/AFL history, the effect was more robust among patients with short and intermediate AF/AFL history, suggesting that treatment with an AAD such as dronedarone should commence at an early stage of disease. Prospective trials are warranted to further investigate the impact of timing of AAD initiation on efficacy and safety among patients with AF/AFL.

## CONFLICT OF INTERESTS

Outside the submitted work, Carina Blomström‐Lundqvist reports consulting fees from Bayer, Boehringer Ingelheim, Boston Scientific, Cardiome, Medtronic, MSD, Pfizer, and Sanofi‐Aventis. Nassir Marrouche reports grants and/or consulting fees from Abbott, Biosense, Boston Scientific, and Medtronic. Stuart Connolly reports consulting fees from Sanofi‐Aventis. Stefan Hohnloser reports consulting fees from Bayer, BMS, Boehringer Ingelheim, Daiichi Sankyo, Pfizer, Sanofi‐Aventis, and Zoll. Andrew Koren was a previous employee of Sanofi. Mattias Wieloch, and Valérie Corp dit Genti are employees of Sanofi.

## Data Availability

The data that support the findings of this study are available from the corresponding author upon reasonable request.
